# Multiplanar MRI-Based Predictive Model for Preoperative Assessment of Lymph Node Metastasis in Endometrial Cancer

**DOI:** 10.3389/fonc.2019.01007

**Published:** 2019-10-09

**Authors:** Xiaojuan Xu, Hailin Li, Siwen Wang, Mengjie Fang, Lianzhen Zhong, Wenwen Fan, Di Dong, Jie Tian, Xinming Zhao

**Affiliations:** ^1^Department of Diagnostic Imaging, National Cancer Center/National Clinical Research Center for Cancer/Cancer Hospital, Chinese Academy of Medical Sciences and Peking Union Medical College, Beijing, China; ^2^CAS Key Lab of Molecular Imaging, Institute of Automation, Chinese Academy of Sciences, Beijing, China; ^3^School of Artificial Intelligence, University of Chinese Academy of Sciences, Beijing, China; ^4^School of Automation, Harbin University of Science and Technology, Harbin, China; ^5^Beijing Advanced Innovation Center for Big Data-Based Precision Medicine, School of Medicine, Beihang University, Beijing, China

**Keywords:** endometrial cancer, lymph node, metastasis, magnetic resonance imaging, radiomics

## Abstract

**Introduction:** Assessment of lymph node metastasis (LNM) is crucial for treatment decision and prognosis prediction for endometrial cancer (EC). However, the sensitivity of the routinely used magnetic resonance imaging (MRI) is low in assessing normal-sized LNM (diameter, 0–0.8 cm). We aimed to develop a predictive model based on magnetic resonance (MR) images and clinical parameters to predict LNM in normal-sized lymph nodes (LNs).

**Materials and Methods:** A total of 200 retrospective patients were enrolled and divided into a training cohort (*n* = 140) and a test cohort (*n* = 60). All patients underwent preoperative MRI and had pathological result of LNM status. In total, 4,179 radiomic features were extracted. Four models including a clinical model, a radiomic model, and two combined models were built. Area under the receiver operating characteristic (ROC) curves (AUC) and calibration curves were used to assess these models. Subgroup analysis was performed according to LN size. All patients underwent surgical staging and had pathological results.

**Results:** All of the four models showed predictive ability in LNM. One of the combined models, Model^CR1^, consisting of radiomic features, LN size, and cancer antigen 125, showed the best discrimination ability on the training cohort [AUC, 0.892; 95% confidence interval [CI], 0.834–0.951] and test cohort (AUC, 0.883; 95% CI, 0.786–0.980). The subgroup analysis showed that this model also indicated good predictive ability in normal-sized LNs (0.3–0.8 cm group, accuracy = 0.846; <0.3 cm group, accuracy = 0.849). Furthermore, compared with the routinely preoperative MR report, the sensitivity and accuracy of this model had a great improvement.

**Conclusions:** A predictive model was proposed based on MR radiomic features and clinical parameters for LNM in EC. The model had a good discrimination ability, especially for normal-sized LNs.

## Introduction

Endometrial cancer (EC) is the most common gynecological malignancy in industrialized countries (1, 2). In China, EC is the second most common malignancy of the female genital tract with patients steadily increasing, especially in high urbanization areas (3). Lymph node metastasis (LNM) is an important risk factor for EC prognosis. Systematic lymphadenectomy is routinely performed according to International Federation of Gynecology and Obstetrics (FIGO). However, there is long-term controversy regarding whether it is necessary for low-risk or stage IA disease (4), as the incidence of LNM is very low in these patients (5). In addition, indiscriminative lymphadenectomy may lead to overtreatment and increase in post-operative complications, including chronic lymphedema, lymphocysts, infection, and nerve/vascular injuries (6).

Several histopathological findings, such as histological subtype, depth of myometrial invasion (DMI), primary tumor diameter (PTD), lymphovascular space invasion, and tumor grade, are known to be risk factors for LNM (4, 5), and researchers proposed various risk-classification models (4, 7). However, most of them are only available post-operatively. Sentinel lymph nodes mapping was proposed to evaluate LNM intraoperatively (8), but the technological dependence on experienced surgeons and relatively high false-negative rates limited its clinical application. Accurate preoperative and non-invasive evaluation of LNM is crucial, which can provide valuable information for prognosis prediction and treatment decision, especially in determining the extent of lymphadenectomy.

Magnetic resonance imaging (MRI) is a routinely used imaging modality for preoperative evaluation of EC. It plays an important role in assessing DMI (9), but its value for LNM assessment remains unsatisfactory, with reported sensitivities of 25–50% (10, 11). Radiomics, as a novel data mining technique, could extract high-dimensional quantitative features from medical images and select reliable features for the establishment of prediction models that could be used in computer-assisted decision support. Some recent researches showed that radiomics had the potential to evaluate therapeutic effects, predict the recurrence and metastasis, predict survival time (12–14), and aid the differential diagnosis of cancers (15). Currently, radiomic investigations in preoperative prediction of LNM showed encouraging achievement (16–18). However, to our knowledge, there is no literature that has determined whether a radiomics-based study would render superior prediction of metastasis in different size groups of LNs, and there has been no study on EC.

The purpose of this study was to investigate the efficacy of multiplanar enhanced MRI-based radiomics for preoperative prediction of metastasis in normal-sized (diameter 0–0.8 cm on MRI) LNs in EC patients.

## Materials and Methods

### Study Design and Participants

This retrospective study with anonymous data was approved by the Ethics Committee of our hospital, and the informed consent requirement was waived.

**Figure 1:**
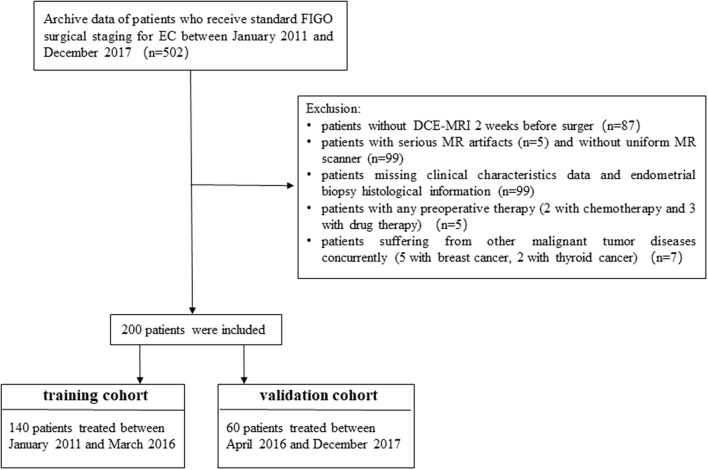
Recruitment pathway for patients in this study.

Two hundred consecutive patients with EC who had been treated between January 2011 and December 2017 were enrolled. Figure 1 shows the patient recruitment pathway. Patients were divided into two independent cohorts: 140 patients treated between January 2011 and March 2016 in the training cohort, and 60 patients treated between April 2016 and December 2017 in the test cohort.

As shown in Table 1, clinical parameters including age, blood serum cancer antigen 125 (CA125) level, preoperative histological type, and differentiation were derived from medical records.

**Table 1:** Baseline characteristics of the training and test cohorts.

	**Training cohort (*****n*** **=** **140)**			**Test cohort (*****n*** **=** **60)**			
	**pN(+)**	**pN(–)**	***P***	**pN(+)**	**pN(–)**	***P***	P*
	***n* = 52**	***n* = 88**		***n* = 15**	***n* = 45**		
Age, years			0.840			0.017	0.077
Mean ± SD	55.271 ± 7.936	55.723 ± 8.382		57.403 ± 6.926	51.730 ± 9.111		
Median (range)	56.000 (28.000–68.000)	56.000 (26.000–80.000)		59.000 (45.000–67.000)	53.000 (35.000–76.000)		
CA125 level (ng/ml),!!! Mean ± SD	86.740 ± 133.348	24.962 ± 23.559	0.002	84.491 ± 100.066	26.772 ± 32.407	0.044	0.539
MR-reported DMI			0.001			0.033	0.524
Less than 50%	19	66		5	35		
More than 50%	33	22		10	10		
MR-reported PTD (mm), Mean ± SD	3.802 ± 2.435	3.929 ± 1.994	0.735	3.758 ± 2.341	3.072 ± 1.535	0.300	0.031
MR-reported tumor staging			<0.001			<0.001	0.659
I	16	68		3	38		
II	3	10		2	2		
III	32	10		10	5		
IV	1	0		0	0		
MR-reported LN status			<0.001			0.367	0.104
cN(+)	17	6		2	2		
cN(–)	35	82		13	43		

*pN(+), pathologically LN positive; pN(−), pathologically LN negative; SD, standard deviation; CA125, cancer antigen 125; DMI, depth of myometrial invasion; PTD, primary tumor diameter; LN, lymph node; cN(+), clinically LN positive; cN(−), clinically LN negative*.

*CA125 level was acquired within 1 week before surgery with a threshold value between 0 and 35 U/ml*.

The P* was derived from the univariate association analyses between each clinical parameter and different cohort.

All MR imaging data were reviewed together by two board-certified radiologists (reader 1 and reader 2) specialized in gynecological tumor imaging with 6 and 20 years of experience. The PTD, DMI, involvement of the cervix, cornua, adnexa, parametrium, and LN status including the size and positive or negative were recorded. Maximal short-axis diameter of LN was measured on delayed phase of dynamic contrast enhanced (DCE) sequence at axial–sectional images (see details below). Patients with pelvic LN > 8 mm or abdominal LN > 10 mm, or with non-homogeneous enhancement and central necrosis on DCE images were regarded as MR report LN-positive (19). The consistency between the two radiologists was assessed by calculating the Cohen's kappa coefficients. Any disagreement was resolved by consultation. Note that LN status was defined by case.

### MR Image Acquisition, Region of Interest Segmentation, and Radiomic Feature Extraction

Before receiving standard FIGO surgical staging, all patients underwent pelvic DCE MRI on two 3.0-T MR scanners (Signa HDxt and Discovery MR750, GE Medical Systems) with 8-channel phased array body coils. Two non-enhanced and one enhanced sequence were obtained and collected for analysis. Detailed scanning parameters are listed in Table 2.

**Table 2:** Detailed acquired parameters in two MR scanners.

	**GE signa excite HD 3.0T**			**GE discovery HD750 3.0T**		
	**Axial T2-fs-FSE^**#**^**	**Sagittal T2-FSE**	Axial 3D-iso-LAVA-XV^*^	**Axial T2-fs-FSE^**#**^**	**Sagittal T2-FSE**	Axial 3D-iso-LAVA-XV^*^
TR/TE	5900/121	3300/130	4.1/1.8	5541/85	4633/120	7.9/4.1
FOV (cm)	40.0	22.0	35.0	40.0	22.0	35.0
Matrix	Freq 320/Phase 256	Freq 320/Phase 256	Freq 350/Phase 350	Freq 320/Phase 256	Freq 320/Phase 256	Freq 350/Phase 350
Slice thickness (mm)	5.0	4.0	1.0	5.0	4.0	1.0
Slice gap	1.0	1.0	0	1.0	0.4	0

# *T2-weighted fat-suppressed fast spin echo (T2-fs-FSE)*.

* *Three-dimensional liver acquisition with volume acceleration DCE with isotropy scanning (3D-iso-LAVA-XV)*.

*TR, repetition time; TE, echo time; FOV, field of view*.

*Enhanced scan was done by injecting gadopentetate dimeglumine (Omniscan, GE Healthcare) into the upper limb vein by using a high-pressure syringe, with a flow rate at 2.0 ml/s and a total dose of 0.2 mmol/kg body weight. A total of 15 phases were obtained post-drug injection with a time interval of 15 s in the sagittal plane, followed by a delayed phase with isotropy axial scanning*.

Tumor volume of interest (VOI), covering the whole tumor volume on each MR image, were manually segmented by reader 1 using ITK-SNAP software (www.itksnap.org, version 3.6.0). Radiomic feature extraction was performed with algorithms implemented in Python (www.python.org, version 3.6.5) (20). Three-dimensional radiomic features were extracted from the corresponding VOIs, including first-order statistics, shape-based, and texture features. More information about the radiomic feature extraction methodology can be found in Supplementary Method 1.

### Surgery and Histopathologic Work-Up

All patients underwent FIGO surgical staging, and accepted template systematic lymphadenectomy. All lymph node specimens were processed and evaluated according to a standard protocol. Histologic analysis of each template lymph node dissection specimen included the following parameters: total number of histologically detected lymph nodes and number of positive nodes in each region as follows: external iliac, internal iliac and obturator, and common iliac. Note that the histopathologic LN status was still considered by case level in our analysis. The 2014 World Health Organization (WHO) classification (21) and the 2009 revised FIGO staging criteria for EC (22) were used for histological diagnosis, grading, and pathological staging.

## Data Analysis

### Feature Selection and Model Construction

Stability analysis of radiomic features between inter-/intra-reader segmentations was firstly carried out. Thirty patients were randomly chosen, and all of their images were segmented separately by the two radiologists, thereinto, reader 1 then re-segmented these images 1 week later. The intraclass correlation coefficients (ICCs) are usually adopted to assess the stability of radiomic features extracted from VOIs delineated by different readers or segmented by the same reader at different times. The radiomic features with ICC >0.75 were retained since they had good agreement between different segmentations.

Then, stability analysis between different versions of MR scanners on radiomic features was carried out. With all the patients randomly assigned to two MR scanners, Mann–Whitney *U* test was used to find out whether a radiomic feature showed statistical difference between different versions of MR scanners in the training cohort. We removed the radiomic features that had significant differences in the two versions of MR scanners, which would improve the generalization capability of our classifier.

**Figure 2:**
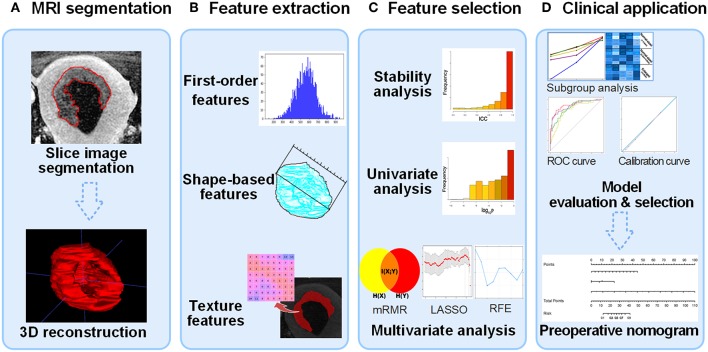
Flow diagram of radiomic model construction. **(A)** MR images segmentation. The tumor region in each MRI slice was manually segmented, and then the whole tumor volume was reconstructed in order to extract 3D radiomic feature. **(B)** Radiomic feature extraction. Three types of radiomic features were extracted from tumor volume. **(C)** Feature selection process including stability, univariate analysis, and multivariate analysis. The construction of Model^C^ starts from univariate analysis. **(D)** Clinical application. After evaluating the four models, an optimal model was selected to plot nomogram for clinical computer-assisted decision support. MRI, magnetic resonance image; 3D, three-dimensional.

Figure 2 shows the workflow of model development and decision-making process for model selection. Four models were constructed, including a clinical model with only clinical parameters (Model^C^), a radiomic model with only radiomic features (Model^R^), and two combined model (Model^CR1^ and Model^CR2^). After model evaluation, the final model was selected to be visualized as a clinical useful preoperative nomogram. The detailed construction processes of the four models were as follows.

#### Model^C^

The original feature set of the Model^C^ consisted of all of the 10 clinical parameters, including age, CA125, tumor pathologic type and differentiation by biopsy, tumor long-axis diameter, DMI, the ratio of tumor infiltration depth to myometrium depth, LN size, and adnexa or other organ involvement, which were all observed on MR images. After feature selection via Mann–Whitney *U* test and the least absolute shrinkage and selection operator (LASSO) method, six features were retained to fit the Model^C^. The logistic regression model was constructed to examine the ability of the clinical parameters in classifying LNM.

#### Model^R^

The stable original feature set of the Model^R^ consisted of 3,040 radiomic features that were dimensionally reduced by stability analysis. Univariate analysis (Mann–Whitney *U* test and chi-square test) was performed to evaluate the difference in LNM status, and the distribution of the *p*-values for the radiomic features is shown in Supplementary Figure 1. The retained significant features were then ranked by minimum redundancy/maximum relevance (mRMR), and the most redundant features were removed. The LASSO method was used to prevent overfitting. Two radiomic features were finally selected to fit the Model^R^. Similarly, logistic regression model was constructed to examine the ability of the radiomic features in classifying LNM.

#### Model^CR1^

All of the clinical parameters and retained 3,040 radiomic features formed the stable feature set. Univariate analysis was performed on this feature set. After removing features with *p*-values > 0.05, we computed the mRMR ranking for the feature set based on the concordance index (23, 24), and the top 5% features were retained. Then, the recursive feature elimination (RFE) method was performed to further select the LNM-related features. The RFE algorithm repeatedly constructed the model and removed the features, depending on the root mean square error of the model by a cross-validation in the training cohort.

#### Model^CR2^

The stable original feature set of the Model^CR2^ consisted of stable radiomic features and clinical parameters except LN size. Univariate analysis was first performed on this feature set. Then, in the multivariable analysis, mRMR and the LASSO method were performed successively. Logistic regression model was constructed to examine the classification ability of the combination of radiomic features and clinical primary lesion information.

### Assessment and Validation of Model Performance

The receiver operating characteristic (ROC) curves were plotted to assess the performance of the four models in both cohorts. Area under ROC curve (AUC) was calculated for quantitative comparison. The model with the highest AUC was selected as the final model. Delong test was used to compare AUCs between the training cohort and test cohort, and a *p*-value > 0.05 indicated that there was no significant difference in AUCs, which ensured that the model had an enough low risk of over-fitting. Calibration curve was plotted to evaluate the agreement between prediction result and gold standard.

In previous research, good effects were gained in predicting the metastasis of an EC-LN larger than 1 cm (25). However, there is no study that ever focused on metastasis prediction on different sized LNs. So, we carried out a subgroup analysis on LN size. Patients were divided into three subgroups according to the LN size measured on MRI, including enlarged LNs with diameter larger than 0.8 cm (>0.8 cm), normal-sized LNs with diameter between 0.3 and 0.8 cm (0.3–0.8 cm), and normal-sized LNs with diameter smaller than 0.3 cm (<0.3 cm). *F*-score $\left(F_{1}=\frac{2Recall\times Precision}{Recall+Precision}\right)$ was calculated in these subgroups, assuming that recall (equivalently, sensitivity, $\frac{TP}{TP+FN}$) and precision (equivalently, PPV, positive predictive value, $\frac{TP}{TP+FP}$) are of equal importance, where TP, FN, and FP represent true positive, false negative, and false positive, respectively. The higher *F*-score synthetically reflects higher sensitivity and higher PPV.

### Clinical Utility of the Final Model

In order to determine the clinical significance of the final model, decision curves were plotted by quantifying the net benefits in the training and test cohort. For the convenience of clinical application, a visualized preoperative nomogram was developed based on the formula exported by the logistic regression of the final model.

### Statistical Analysis

In this study, statistical analysis programs were completed by R software (version 3.5.0; https://www.r-project.org). All statistical hypothesis tests were two-sided, and *p*-values < 0.05 were considered significant.

## Results

### Patient Characteristics

The clinical and pathological characteristics in the two cohorts are shown in Tables 1, 3, respectively. Pathologically LN positive (pN+) patients formed 37.14% (52/140) and 25.00% (15/60) of the training and test cohorts, respectively, and there was no significant difference between them (*p*-value = 0.133, χ^2^ test). The clinical parameters age and CA125 had no differences between the two cohorts (*p*-value = 0.077 and 0.539 respectively, Mann–Whitney *U*-test). In total, sensitivity and specificity were 28.36% (19/67) and 93.98% (125/133) according to the MR report LN status after consensus within two radiologists in our study. Also, the judgments by two radiologists on MRI were basically stable (sensitivity was 0.642 and 0.552, and the specificity was 0.917 and 0.940, respectively). The inconsistency of judgment was resolved by consultation. The Cohen's kappa coefficients to test consistency of the main MR indicators evaluated by the two radiologists are listed in Supplementary Table 1.

**Table 3:** Pathological characteristics of the patients in our study.

	**Training cohort (*n* = 140)**	**Test cohort (*n* = 60)**	***P***
**Surgically histological type**, ***n*** **(%)**			0.602
Endometrioid	112 (80%)	47 (78.33%)	
Non-endometrioid	28 (20%)	13 (21.67%)	
**Histological grade**, ***n*** **(%)**			0.041
Well-differentiated	67 (47.86%)	17 (28.33%)	
Moderately differentiated	52 (37.14%)	37 (61.67%)	
Poorly differentiated	21 (15.00%)	6 (10.00%)	
**Pathological N stage**, ***n*** **(%)**			0.133
pN–	88 (62.86%)	45 (75.00%)	
pN+	52 (37.14%)	15 (25.00%)	
**Pathological staging**, ***n*** **(%)**			0.250
pI	68 (48.57%)	38 (63.33%)	
pII	16 (11.43%)	6 (10.00%)	
pIII	50 (35.71%)	15 (25.00%)	
pIV	6 (4.29%)	1 (1.67%)	

### Feature Selection and Model Construction

In total, 1,393 radiomic features were extracted from each of the three MR scanning sequences. Then, 4,179 radiomic features were reduced to 3,040 by stability analysis.

**Figure 3:**
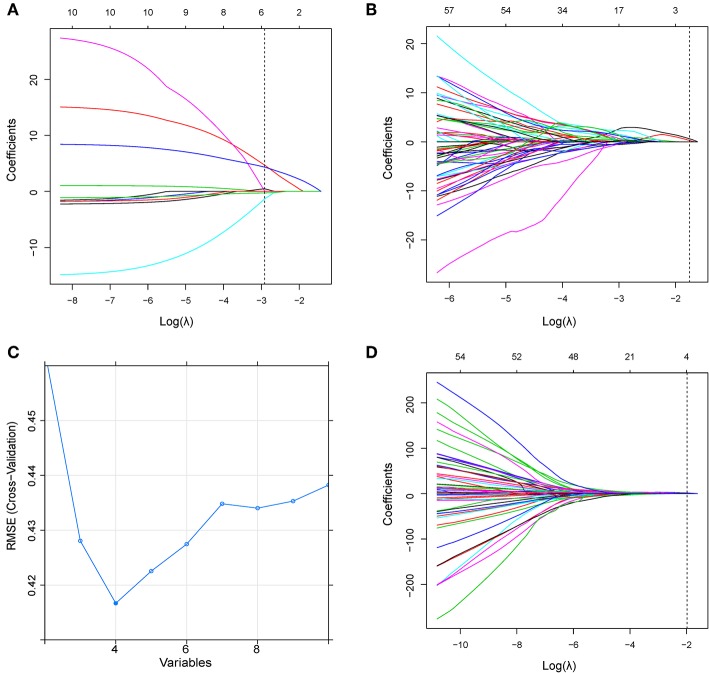
**(A)** LASSO coefficient profiles of the clinical parameters in Model^C^. According to the 1 standard error of the minimum criteria (the 1-SE criteria), the dotted line was plotted at the selected log(λ) (−2.914) via 10-fold cross-validation. **(B)** LASSO coefficient profiles of the radiomic features in Model^R^. A log(λ) value of −1.750 was chosen (10-fold cross-validation, 1-SE criteria). **(C)** Feature selection using the RFE method in Model^CR1^. The rank of feature importance was obtained using the random forest method; RFE built the model continuously by eliminating the lower ranking feature. The RMSE was used to select the optimal feature set in a 10-fold cross-validation. **(D)** LASSO coefficient profiles of the combined feature set in Model^CR2^. A log(λ) value of −1.983 was chosen (10-fold cross-validation, 1-SE criteria). RFE, recursive feature elimination; RMSE, root mean square error.

In Model^C^, six clinical parameters were selected including CA125, tumor differentiation by biopsy, DMI, the ratio of tumor infiltration depth to myometrium depth, LN size, and adnexa involvement, which were all observed on MR. In Model^R^, two radiomic features were selected including correlation and HGLE. In Model^CR1^, four risk factors including two clinical parameters (CA125 and LN size) and two radiomic features (correlation and HGLE) were used to build the prediction model (Figure 3) (13). The two radiomic features were extracted from the delayed phase of the 3D-Iso-LAVA and sagittal T2WI FSE, respectively. In Model^CR2^, the LN size was removed and the same other three indicators (CA125, correlation, and HGLE) were selected. The detailed calculation formulas for Model^CR1^ and Model^CR2^ were given in Supplementary Method 2.

### Assessment of Predictive Models

**Figure 4:**
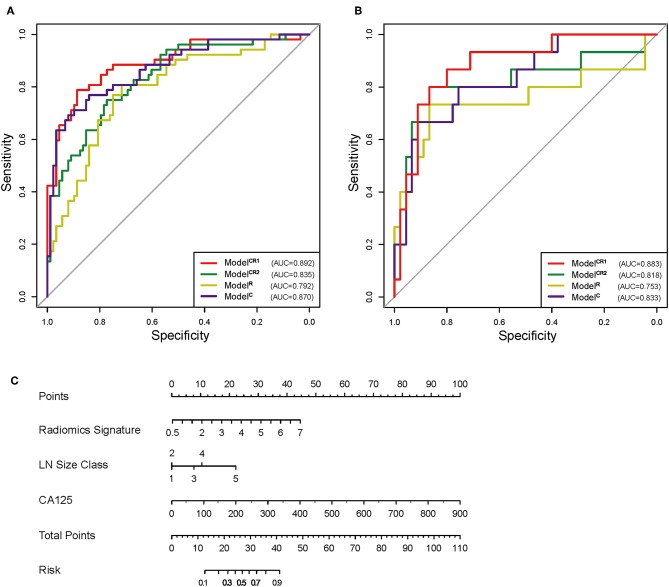
**(A)** ROC of the four models in training cohort. **(B)** ROC of the four models in test cohort. **(C)** Preoperative nomogram of Model^CR1^. ROC, receiver operating characteristic.

**Figure 5:**
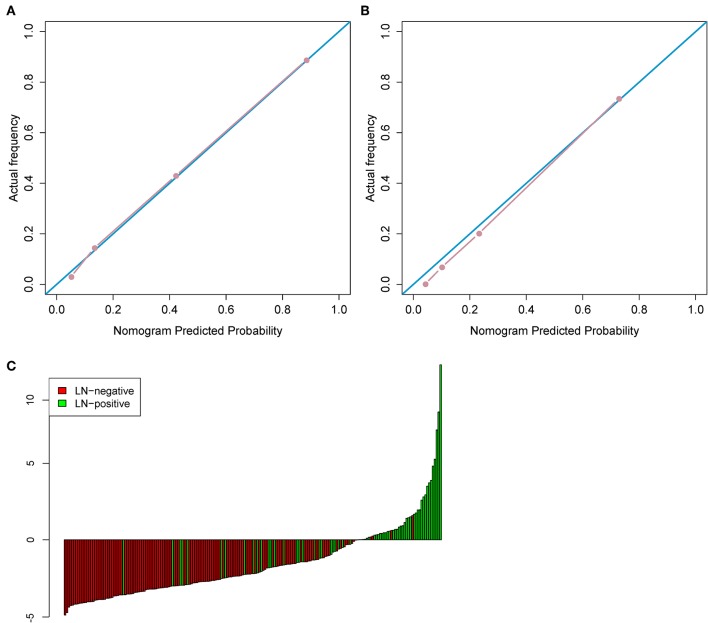
**(A)** The calibration curve in the training cohort. **(B)** The calibration curve in the test cohort. **(C)** Patient risk score output by Model^CR1^, while red bars show scores for those who were pathologically LN(–).

Model^CR1^ showed a significant ability in detecting pN+ with an AUC of 0.892 [95% confidence interval [CI]: 0.834–0.951] in the training cohort and an AUC of 0.883 (95% CI: 0.786–0.980) in the test cohort. Nomogram (Figure 4C) was established for Model^CR1^. The *p* values calculated from Delong tests were 0.875, 0.8416, 0.7008, and 0.5865 for Model^CR1^, Model^CR2^, Model^R^, and Model^C^, respectively, indicating that there were no significant differences in AUCs between the training cohort and test cohort for each model. Performances of the four models in the training and test cohort are shown in Figures 4A,B. Based on the threshold determined by Youden's index in the training cohort, we used net reclassification index (NRI) to analyze the improvement brought by Model^CR1^ compared with other models. The results showed that Model^CR1^ outperformed Model^R^ (NRI = 0.306, *P* < 0.001), Model^C^ (NRI = 0.134, *P* = 0.010), and Model^CR2^ (NRI = 0.090, *P* = 0.077). Meanwhile, Model^CR1^ also significantly surpassed MR reports by radiologists (NRI = 0.489, *P* = 0.006). Besides, the calibration curves were plotted in both cohorts for further performance evaluation of Model^CR1^ (Figures 5A,B). Calibration curves show good fitness for probability of LNM (Hosmer–Lemeshow test, *p*-value = 0.961 in the training cohort, 0.803 in the test cohort). Figure 5C shows patients' risk scores calculated from Model^CR1^, intuitively indicating its high classification ability.

**Figure 6:**
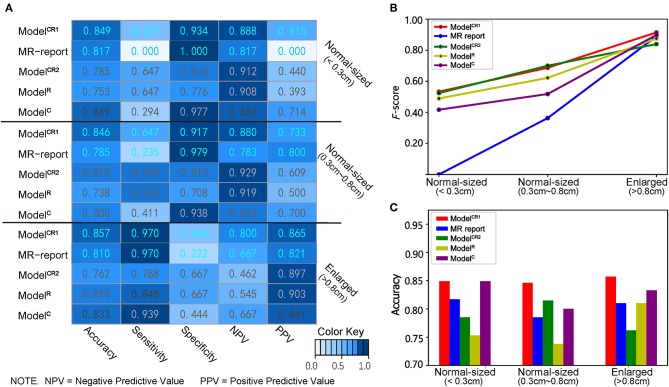
**(A)** Heatmap of showing the classifiers' performance in different LN size subgroups. A deeper blue indicates a larger value. **(B)** Line chart of *F*-score, **(C)** histogram of accuracy in three subgroups. Red, blue, green, yellow, and purple lines and boxes, respectively, represent Model^CR1^, MR report, Model^CR2^, Model^R^, and Model^C^.

As shown in Figure 6A, in the subgroup of enlarged LNs, Model^CR1^ achieved the highest sensitivity of 0.970, equal to that predicted by MR report. In the subgroup of normal-sized LNs (0.3–0.8 cm), Model^CR1^ displayed the highest accuracy of 0.846 and a sensitivity of 0.647, which far surpassed the MR report (accuracy, 0.785; sensitivity, 0.235). In the subgroup of normal-sized LNs (<0.3 cm), ModelCR1 showed the best accuracy of 0.849 and a moderate sensitivity of 0.471, however, still greatly outperforming the MR report (accuracy, 0.817; sensitivity, 0.000). Meanwhile, *F*-score and accuracy in three subgroups are shown in Figures 6B,C, respectively. The highest *F*-score and most powerful accuracy of ModelCR1 were reflected among the five predictive models.

## Discussion

In the present study, we developed four predictive models based on multiplanar DCE MR images and clinical parameters for LNM in EC patients. Model^CR1^, which consisted of radiomic features, LN size, and CA125, showed the best discrimination ability, especially in patients with normal-sized LNs (diameter, 0–0.8 cm on MRI) and the sensitivity was greatly improved compared with the routine MR reports. The high *F*-scores indicated that while the sensitivity increased significantly, the PPV remained high.

A non-invasive and convenient preoperative assessment for LNM is crucial for EC treatment decision and prognosis prediction. Patients' data from preoperative procedures such as MRI, biopsy, and CA125 have been studied to assess LNM in recent years. MRI still remained the cornerstone in LN assessment in EC, showing satisfactory specificity but relatively low sensitivity. The combination of relative apparent diffusion coefficient (ADC) value and LN size was reported to result in a significant increase in sensitivity from 25 to 83% compared with conventional MRI (26); however, there have been conflicting reports in the literature regarding the detection of LNM at diffusion-weighted MR imaging (DWI). Nakai et al. (27) used 1.5-T MRI to evaluate nodal ADC values in gynecologic malignancies and were unable to differentiate benign from malignant LNs. Wang et al. (28) proposed a tumor biomarker predictive method by combining human epididymis secretory protein 4 (HE4) and CA125, achieving a high sensitivity of 94.1% but a low specificity of 30.7%. Notably, there is no clearly defined HE4 cutoff value for EC at present. In our study, we incorporated CA125 in our models, which was more generally accepted than HE4. Kang et al. (29) developed a low-risk prediction model for LNM based on MRI and serum CA125 data in endometrioid-type EC patients, and obtained sensitivity and specificity of 84.9 and 55.5%, respectively. Here, three MRI parameters including DMI, LN enlargement, and extension beyond uterine corpus were identified to be independent risk factors for LNM. In our study, we obtained CA125 and MR report LN size as risk factors for EC LNM prediction, which was similar to that result, and showed good discrimination ability on both cohorts and different LN size subgroups, especially for those normal-sized LNs, which previous researches had not yet focused on.

In our study, we collected and analyzed all available preoperative clinical parameters and established four prediction models. We aimed to determine the prediction efficiency of different models compared with the MR report in different sized LN subgroups. MRI uses several common morphological criteria in differentiating benign from malignant nodes (30) but nodal size still remains the commonly accepted standard. Low sensitivity is a recognized limitation when nodal size criteria are used on cross-sectional imaging, especially for normal-sized nodes due to limited spatial resolution. In this study, with node size gradually decreasing, the MR report and Model^C^ showed a decreasing sensitivity, whereas the Model^R^ and Model^CR2^ were more stable because of the high sensitivity in each sized LN subgroup (Supplementary Figure 2). The performance of the above classifiers confirmed our thoughts: When the LN was normal sized on MRI, combining LN size in classifiers could improve prediction accuracy but greatly reduce sensitivity. It is already accepted that normal-sized LNs may also contain metastases (31). The results of the MR report rely too much on LN size so that when LN size is normal on MRI, the sensitivity becomes very low. The concept remains the same when LN size is enlarged (>0.8 cm), then the specificity becomes very low. This can be due to the fact that it is usually difficult to differentiate enlarged nodes because of benign pathology, such as infection, granulomatous disease, and reactive hyperplasia vs. malignant disease (30). LN size was not a significant predictor for the fusion model based on radiomics, although it could improve the predictive accuracy. This may indicate that there was enough information contained in the primary tumor region that could detect LNM. It is feasible to predict LNM status without the dependence on information of LNs.

Although Model^CR1^ showed a slightly lower sensitivity than Model^R^ and Model^CR2^ in the normal-sized group, its accuracy in each group is the highest, and the *F*-score with normal-sized LNs is greatly improved. Therefore, it was proposed as the optimal prediction model. To our knowledge, this is the first subgroup analysis on different sized LNs with preoperative nomogram study in EC.

Due to a variety of MR scanner parameter settings and scanner models, it is difficult to guarantee different scanners with exactly the same imaging quality, thus making it difficult to ensure the stability of radiomic features. By eliminating the radiomic features sensitive to scanner models and parameter settings in the training cohort, the radiomic model generalization ability can be improved. The radiomic texture features (correlation and HGLE) selected in Model^CR1^, Model^CR2^, and Model^R^ reflected two kinds of heterogeneity of VOI with a Pearson correlation coefficient of 0.095. Correlation shows the linear dependency of gray-level values to the corresponding voxels in the gray-level cooccurence matrix (GLCM) of MRI. HGLE is a measure of the proportion of areas with higher gray values in the tumor. These two radiomic features indicated that the extent of heterogeneity of tumor is associated with LNM. The more heterogeneous the tumor, the higher the risk of LNM.

The limitations of the present study include two aspects. First, there was no external validation. Multicenter investigation with a larger dataset was needed to further validate the generalization ability of our model. Second, genomic information was not yet incorporated into our models. A combination of gene marker panels and radiomic features will be promising in evaluation of EC.

In conclusion, our study presented a predictive model based on multiplanar contrast enhanced MR images and incorporated both the radiomic features and clinical parameters, which showed good predictive accuracy for preoperative LNM in EC, especially in patients with normal-sized LNs.

## Data Availability Statement

The datasets generated for this study are available on request to the corresponding author.

## Ethics Statement

The studies involving human participants were reviewed and approved by National Cancer Center/Cancer Hospital, Chinese Academy of Medical Sciences and Peking Union Medical College. Written informed consent for participation was not required for this study in accordance with the national legislation and the institutional requirements.

## Author Contributions

XX, HL, XZ, DD, JT, MF, and SW: conception and design. XX, HL, WF, and LZ: collection and assembly of data. HL, XX, DD, XZ, and MF: data analysis and interpretation. All authors: manuscript writing and final approval of manuscript.

### Conflict of Interest

The authors declare that the research was conducted in the absence of any commercial or financial relationships that could be construed as a potential conflict of interest.

## Abbreviations

LNM: lymph node metastasis
EC: endometrial cancer
LN: lymph node
AUC: area under the curve
MRI: magnetic resonance imaging
IGO: International Federation of Gynecology and Obstetrics
DMI: depth of myometrial invasion
A125: cancer antigen 125
PTD: primary tumor diameter
WHO: World Health Organization
DCE: dynamic contrast enhanced
VOI: volume of interest
ICC: intraclass correlation coefficient
mRMR: minimum redundancy/maximum relevance
RFE: recursive feature elimination
HGLE: high gray-level emphasis
LASSO: least absolute shrinkage and selection operator
ROC: receiver operating characteristic
PV: positive predictive value
TP: true positive
FN: false negative
FP: false positive
pN+: pathologically LN positive
3D-iso-LAVA-XV: three-dimensional liver acquisition with volume acceleration DCE with isotropy scanning
CI: confidence interval
ADC: apparent diffusion coefficient
DWI: diffusion-weighted MR imaging
HE4: human epididymis secretory protein 4
GLCM: gray-level cooccurrence matrix.
