# Supplementary material for: Multiplanar MRI-Based Predictive Model for Preoperative Assessment of Lymph Node Metastasis in Endometrial Cancer
Source: Front Oncol. 2019 Oct 9;9:1007. doi: 10.3389/fonc.2019.01007 (PMC6794606; doi:10.3389/fonc.2019.01007)
Supplement: Supplementary file 1 [file Data_Sheet_1.pdf]

Supplementary Material

Supplementary Table S1. Consistency between the two radiologists with MR indicators.
Supplementary Figure S1. The distribution of the *p*-value for the original feature set.
Supplementary Figure S2. Heatmap of showing the four models' sensitivity with a change in LN size.
Supplementary Method S1. Radiomic features.
Supplementary Method S2. The formulas generated from Model^{CR1} and Model^{CR2}.

Supplementary Table S1. Consistency between the two radiologists with MR indicators.

MR indicators	Kappa value
involvement of the cervix	0.855
involvement of the cornua	0.957
involvement of the adnexa	0.835
LNM in internal iliac and obturator	0.813
LNM in common iliac	0.656
LNM in external iliac	0.655

The kappa statistic was interpreted as follows: less than 0, no agreement; 0–0.20, slight agreement; 0.21–0.40, fair agreement; 0.41–0.60,moderate agreement; 0.61–0.80, substantial agreement; and 0.81–1.0, almost perfect agreement.

Supplementary Figures

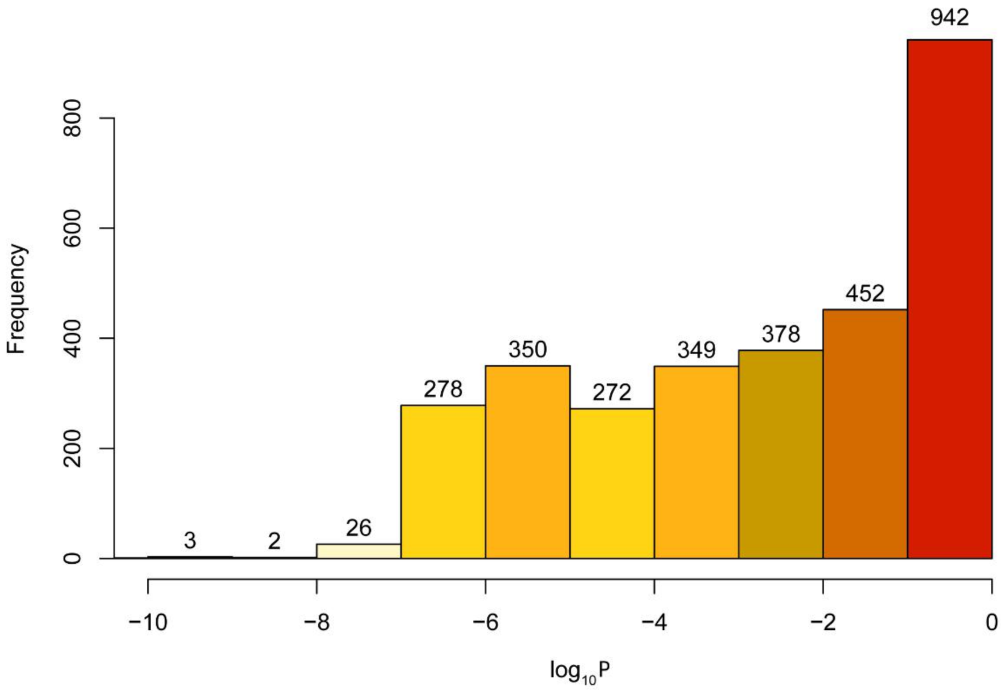

Supplementary Figure S1 The distribution of the *p*-value for the original feature set. The Mann-Whitney U-test was used for continuous variables, and the chi-square test was used for discrete variables.

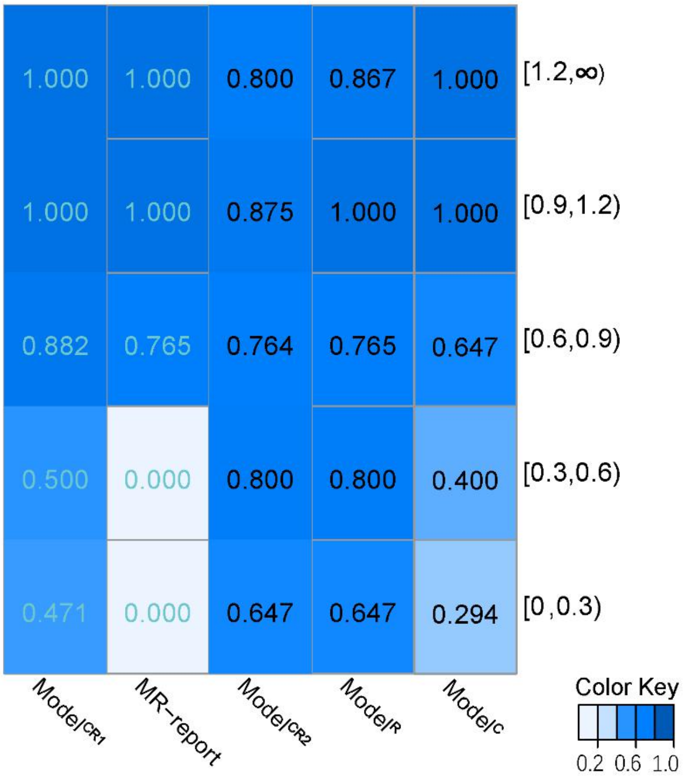

Supplementary Figure S2 More detailed heatmap of showing the four models' sensitivity with a change in LN size. A deeper blue indicates a larger value. It exhibits the sensitivities in different sizes of scattered LN in more detail. With the decrease in LN size, the sensitivity changed dramatically in the MR-report and Model^{C}, and less in the Model^{R}, Model^{CR1} and Model^{CR2}.

Supplementary Methods

Supplementary Method S1. Radiomic features

In this study, a total of 4179 candidate radiomic features were generated from axial T2WI FS-FSE, sagittal T2WI-FSE and delayed phase of 3D-Iso-LAVA[1], including 809 first-order statistics features, 39 shape-based features and 3330 texture features. Apart from shape-based features, all radiomic features could also be calculated from derived images, obtained by applying a variety of filters. The derived images usually showed greater ability in pattern classification.

First-order statistics features based on the gray level histogram describe the distribution of MRI voxel intensities. The basic first-order statistics features include energy, total energy, entropy, minimum, 10th percentile, 90th percentile, maximum, mean, median, interquartile range, range, mean absolute deviation, robust mean

absolute deviation, root mean squared, standard deviation, skewness, kurtosis, variance, and uniformity.

Shape-based features describe the tumor shape in three dimensions. That is, shape-based features are only affected by the ROI shape; therefore, filters are not required. The basic shape-based features include volume, surface area, surface area to volume ratio, sphericity, compactness, spherical disproportion, maximum 3D diameter, maximum 2D diameter (slice), maximum 2D diameter, major axis, minor axis, least axis, elongation, and flatness.

Texture features reflect the surface structure of the tumor surface with slow or periodic changes, expressed by the gray level distribution of the neighborhood of the voxel. The features used to fit the model in this study were all texture features ("ce_wavelet.LLL_glcm_Correlation" and "t2sag_wavelet.HHL_gldm_SmallDependenceHighGrayLevelEmphasis"). The $(i,j)^{th}$ element of GLCM represents the number of times the combination of levels i and j occur in two pixels in the image. The specific calculation formula is as follows (1-1). Small dependence high gray level emphasis (SDHGLE) measures the joint distribution of large dependence with higher gray-level values in the gray level dependence matrix (GLDM). It reflects the proportion of darker and smaller areas of the ROI. The specific calculation formula is as follows (1-2). The feature "t2sag_wavelet.HHL_glszm_SmallAreaHighGrayLevelEmphasis (SAHGLE)" selected in Model^{CR2} was strongly correlated with the above feature with a Pearson correlation coefficient of 0.800. They have nearly identical meaning in MRI, and they were collectively called HGLE in this study.

$$\text{Correlation} = \frac{\sum_{i=1}^{N_g}\sum_{j=1}^{N_g} p(i,j)ij - \mu_x\mu_y}{\sigma_x(i)\sigma_y(j)} \qquad (1\text{-}1)$$

where

$p(i,j)$	is the normalized co-occurence matrix
N_g	is the number of discrete intensity levels in the image
σ_x	is the standard deviation of p_x
μ_x	is the mean gray level intensity of p_x

$$\text{SDHGLE} = \frac{\sum_{i=1}^{N_g}\sum_{j=1}^{N_s} \frac{P(i,j)i^2}{j^2}}{N_z} \qquad (1\text{-}2)$$

where

$p(i,j)$	is the normalized dependence matrix
N_g	is the number of discreet intensity values in the ROI
N_s	is the number of discreet dependency sizes in the ROI

N_z is the number of dependency zones in the ROI

Supplementary Method S2. The formula generated from the logistic regression model

Formula in Model[CR1].

$$\text{score}^{CR1} = 1 \times \text{Radiomics signature}^1 + 0.0174 \times \text{CA125} + 1.244 \times \text{IF}(\text{MRreportLNS} = 3) + 1.613 \times \text{IF}(\text{MRreportLNS} = 4) + 3.519 \times \text{IF}(\text{MRreportLNS} = 5)$$

where

$\text{Radiomics signature}^1$ is the linear weighted sum of the radiomic features, which is equal to

$$4.744 \times \text{ce.wavelet.LLL.glcm.Correlation} + 5.758 \times \text{t2sag.wavelet.HHL.gldm.SDHGLE}$$

$MRreportLNS = n$ is the size of LNs in the MR-report, where n=3 means 0.6-0.9cm, n=4 means 0.9-1.2cm, and n=5 means the size exceeds 1.2cm. Note that n=1 and n=2 mean 0-0.3cm and 0.3-0.6cm respectively, and they do not appear in the formula because the coefficient is 0.

Formula in Model[CR2].

$$\text{score}^{CR2} = 1 \times \text{Radiomics signature}^2 + 0.0182 \times \text{CA125}$$

where

$\text{Radiomics signature}^2$ is the linear weighted sum of the radiomic features, which is equal to

$$4.261 \times \text{ce.wavelet.LLL.glcm.Correlation} + 4.720 \times \text{t2sag.wavelet.HHL.glszm.SAHGLE}$$

References for supplements

[1] van Griethuysen JJM, Fedorov A, Parmar C, Hosny A, Aucoin N, Narayan V, et al. Computational radiomics system to decode the radiographic phenotype. Cancer research. 2017;77:e104-e107
